# Combined Facile Synthesis, Purification, and Surface Functionalization Approach Yields Monodispersed Gold Nanorods for Drug Delivery Applications

**DOI:** 10.1002/ppsc.202300043

**Published:** 2023-09-05

**Authors:** Shunping Han, Khuloud T. Al‐Jamal

**Affiliations:** ^1^ Institute of Pharmaceutical Science Faculty of Life Sciences & Medicine King's College London Franklin‐Wilkins Building, 150 Stamford Street London SE1 9NH UK

**Keywords:** cytotoxicity, gold nanorods, optimization, purification, synthesis

## Abstract

Synthesizing gold nanorods (AuNRs) by seed‐mediated growth method results in the presence of undesired size and shape particles by‐products occupying 10–90% of the population. In this study, AuNRs are synthesized by the seed‐mediated growth method using cetyltrimethylammonium bromide (CTAB) as a surfactant. AuNRs with redshifted longitudinal localized surface plasmon resonance (LLSPR) peak, localized in the biological “transparency window” (650–1350 nm), are synthesized after optimizing seed solution, silver nitrate solution, and hydrochloric acid solution volumes, based on the published protocols. A two‐step purification method, dialysis followed by centrifugation, is applied to remove excess CTAB and collect LLSPR‐redshifted AuNRs with high rod purity (>90%). CTAB is subsequently exchanged with polyethylene glycol (PEG) to improve AuNRs biocompatibility. PEGylated AuNRs are confirmed innocuous to both SN4741 cells and B16F10 cells by the modified MTT assay and the modified lactate dehydrogenase (LDH) assay up to 1 nm and 24 h incubation. In this study, a combined facile synthesis, purification, and surface functionalization approach is proposed to obtain water‐dispersible monodispersed AuNRs for drug delivery applications.

## Graphical abstract

The seed‐mediated growth method is applied to generate gold nanorods (AuNRs). By controlling the growth procedure, the longitudinal localized surface plasmon resonance of the synthesized AuNRs is localized into the biological “transparency window”. A combinational purification method is applied to remove excessive cetyltrimethylammonium bromide (CTAB) and further improve rod shape purity. Surface functionalization with polyethylene glycol (PEG) results in improved biocompatibility compared to CTAB‐AuNRs.

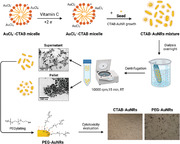

## 1 Introduction

Gold nanorods (AuNRs) owing to their tunable size and size‐dependent surface plasmon resonance (SPR) are of interest for numerous applications, including sensing, photonics, bioimaging, biomedicine, and nanoelectronics.^[^ ^1^, ^2^ ^]^ The cylindrical conformation of AuNRs makes it possible to produce two surface plasmon resonances: longitudinal localized surface plasmon resonance (LLSPR) and transverse localized surface plasmon resonance (TLSPR). The TLSPR is relatively insensitive to the nanorod size and is usually centered around 520 nm, while the LLSPR contributes to the specific absorbance wavelength and is mainly dimension‐dependent. When the aspect ratio (long axis/short axis, AR) of AuNRs increases, the LLSPR shifts deeper into the tissue transparent near‐infrared (NIR) region, 650–1350 nm, also known as the biological “transparency window”^[^ ^3^, ^4^ ^]^, in which the smallest blood and tissue attenuation occurs. This optical absorption property of AuNRs makes them attracting photo‐absorbing agents for photoacoustic imaging (PAI) and photothermal therapy (PTT) applications.^[^ ^4^ ^]^

Synthesis of AuNRs with the seed‐mediated growth method using cetyltrimethylammonium bromide (CTAB) as a surfactant is the most conventional approach for its cost‐effectiveness and lack of need for specialized equipment.^[^ ^5^ ^]^ A disadvantage to this method is the production of particles impurities with undesired size and shape which sometimes occurs at unacceptable levels. Several protocols for AuNR synthesis have been published with batch‐to‐batch variations in LLSPR peak wavelength and degree of impurities ranging from 10% to 90%.^[^ ^6^, ^7^ ^]^ Centrifugation is the most used method to collect AuNRs after synthesis. This method of purification however does not discriminate between the desired particles and those defined as impurities.

Here in this work, we report an optimized AuNR synthesis protocol based on the seed‐mediated growth method and CTAB as a surfactant. We have combined this with a dialysis/centrifugation purification and polyethylene glycol (PEG)‐thiol functionalization methods which altogether resulted in AuNRs with redshifted LLSPR peak, high purity and negligible *in vitro* toxicity suitable for biological applications.

## 2 Results

### 2.1 CTAB‐AuNRs with a Single, Dominant, and Redshifted LLSPR Peak were Synthesized

#### 2.1.1. Seed Solution to Growth Solution Volume Ratio

The seed‐mediated growth method due to their benefits including the simplicity of the experimental procedure and relatively high particle yield^[^ ^8^, ^9^ ^]^ was applied in this study to obtain AuNRs using CTAB as a surfactant. Although the AuNR synthesis protocols using seed‐mediated growth methods are widely established (the chemical mechanism is shown in Figure S1, Supporting Information), the batch‐to‐batch, lab‐to‐lab, or person‐to‐person variation makes the generated LLSPR peak hugely variable. The published protocol was therefore modified to home‐make CTAB‐AuNRs with red‐shifted LLSPR peak. The volume ratio of seed solution to growth solution can strongly influence the LLSPR of CTAB‐AuNRs prepared by the seed‐mediated growth method.^[^ ^10^ ^]^ While fixing the growth solution volume, 3 seed solution volumes were tested based on published reports^[^ ^11^ ^]^ (Table S1, Supporting Information). Increasing the seed solution volume from 90 µL to 180 µL resulted in the dominant LLSPR peak blueshift from 658 nm to 622 nm while increasing the volume to 360 µL resulted in a redshift to 696 nm. A seed solution volume of 360 µL which resulted in the largest wavelength in LLSPR peak of 696 nm (CTAB‐AuNRs‐1c, Table S1) was therefore chosen for subsequent synthesis parameters optimization (Figure S2A, Supporting Information).

#### 2.1.2. AgNO_3_ amount

AgNO_3_ is responsible for the anisotropic growth of CTAB‐AuNRs.^[^ ^12^ ^]^ By investigating different amounts of AgNO_3_ (Table S1, Supporting Information), the smallest volume of AgNO_3_ tested (0.90 mL) resulted in a single and dominated LLSPR peak at 704 nm observed in CTAB‐AuNRs‐2a (Figure S2B, Supporting Information). Increasing the volume of AgNO_3_ to 2.25 and 3.60 mL resulted in the multiple UV–vis‐NIR spectra peaks with dominant LLSPR peaks redshifted to 835 and 935 nm, respectively. Despite 3.60 mL achieved the most red‐shifted LLSPR peak (CTAB‐AuNRs‐2c), the multiple peaks suggested shape impurities which required further method optimization to obtain a single, narrow and dominant red‐shifted LLSPR peak.

#### 2.1.3. Presence of Acidifying Agent

**Figure 1:**
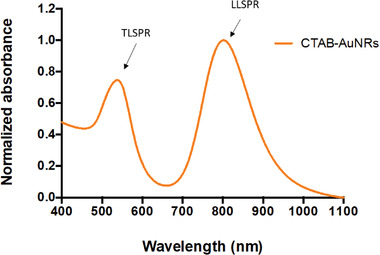
Normalized UV‐vis‐NIR spectra of CTAB‐AuNRs. AuNRs synthesized with optimal volumes generated a single, dominant and redshifted LLSPR peak > 800 nm.

It is hypothesized that HCl addition to the growth solution can slow down AuNRs generation rate by weakening Vitamin C reducing power which could slow lateral surfaces growth rate and facilitate the formation of AuNRs with a high AR and improved shape purity.^[^ ^13^ ^]^ To study this experimentally, different volumes of 1 M HCl solution were added to the growth solution (Table S1, Supporting Information) while fixing seed solution and AgNO_3_ volume to 360 µL and 3.60 mL, respectively. Increasing HCl volumes redshifted of LLSPR peaks from 670 to 801 nm (Figure S2C, Supporting Information). Additionally, a single and dominant LLSPR peak (Figure S2C, Supporting Information, CTAB‐AuNRs 3b‐e) was observed compared to the multiple plasmon bands observed in the absence of acidification (Figure S2C, Supporting Information, CTAB‐AuNRs‐3a). This confirmed that HCl addition helped producing AuNRs with redshifted LLSPR peak and improved shape purity. The methodology used to produce CTAB‐AuNRs‐3e that exhibit a single, dominant, and largest redshifted LLSPR peak (**Figure** **1**) was used to produce CTAB‐AuNRs in this study. To summarize, the following conditions were used to synthesize AuNRs with single and dominant LLSPR peak of > 800 nm: seed solution, AgNO_3_ solution, and HCl solution volumes of 360 µL, 3.60 mL, and 1.44 mL, respectively. Subsequent purification procedure was introduced to enforce AuNRs shape purity and further redshift of LLSPR peak.

### 2.2 A Two‐Step Purification Method Removed Excess CTAB and Further Improved the Shape Purity of CTAB‐AuNRs

**Figure 2:**
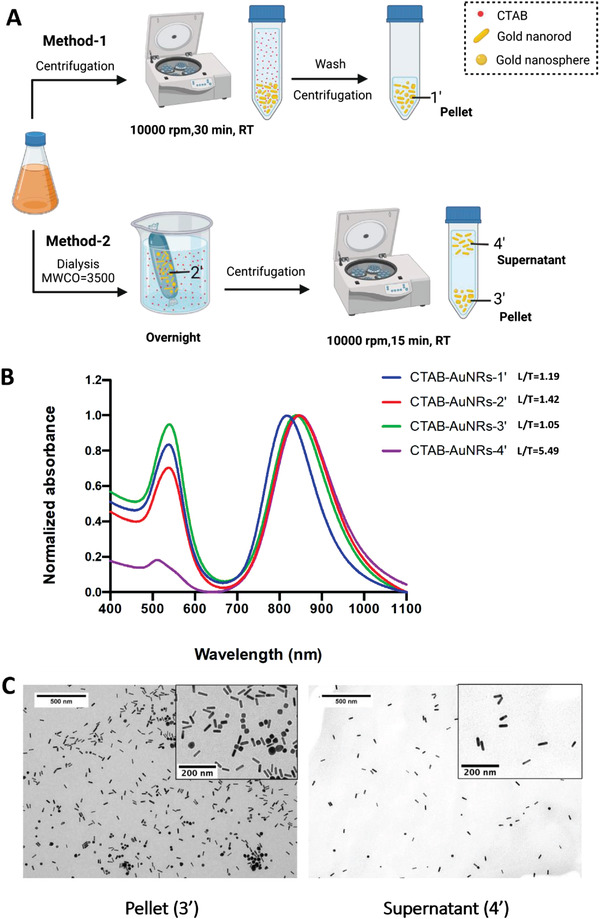
Purification and characterization of CTAB‐AuNRs. A) Purification methods after CTAB‐AuNRs synthesis. Method‐1: sample was purified by centrifugation (10 000 rpm, 30 min, RT). Method‐2: sample was purified by a combinational method firstly by an overnight dialysis (MWCO = 3500) followed by centrifugation (10,000 rpm, 15 min, RT). B) Normalized UV‐vis‐NIR spectra of CTAB‐AuNRs obtained from the different fractions. CTAB‐AuNRs‐1′: pellets obtained from method‐1; CTAB‐AuNRs‐2′: suspension obtained after dialysis from method‐2; CTAB‐AuNRs‐3′: pellets obtained from method‐2; CTAB‐AuNRs‐4′: supernatant obtained from method‐2 after centrifugation. L/T: LLSPR to TLSPR intensity ratio. C) TEM images of CTAB‐AuNRs‐3′ and CTAB‐AuNRs‐4′. Scale bar (zoomed‐in) = 200 nm.

AuNRs contain excess CTAB which should be eliminated since CTAB as a quaternary surfactant exhibits an undesirable toxicity profile. Therefore, after synthesis, the mixtures were purified by one of two methods to remove excess CTAB and collect CTAB‐AuNRs with further redshifted LLSPR peak, as shown in **Figure** **2**A. CTAB‐AuNRs (CTAB‐AuNRs‐1′) purified by Method‐1 (centrifugation only, 10 000 rpm, 30 min, RT) has an LLSPR peak and an intensity ratio of LLSPR peak to TLSPR peak (L/T ratio) of 817 nm and 1.19, respectively (Figure 2B).

An additional dialysis step to remove excess unbound CTAB was introduced in Method‐2. When using Method‐2, CTAB‐AuNRs after dialysis (CTAB‐AuNRs‐2′) showed a redshift of the LLSPR peak from 817 to 847 nm with L/T ratio of 1.42 (Figure 2B). A gentle centrifugation step (10 000 rpm, 15 min, RT) of the dialyzed sample could successfully separate the rod‐shaped particles referred to as CTAB‐AuNRs‐4′ (supernatant AuNRs fractions) from the highly impure sample referred to as CTAB‐AuNRs‐3′ (pelleted AuNRs). The LLSPR peaks and L/T ratio of CTAB‐AuNRs‐3′ and CTAB‐AuNRs‐4′ were 841/848 nm and 1.05/5.49, respectively. The fivefold increase in L/T ratio for the supernatant fraction suggests presence of more rod‐shaped AuNRs compared to spherical particles enriching the pellet fraction. The hydrodynamic size and zeta potential of CTAB‐AuNRs in different fractions determined by nanoparticle tracking analysis (NTA) and Zetasizer Nano series were shown in Figure S3 and Table S2 (Supporting Information). Compared with CTAB‐AuNRs‐3′, CTAB‐AuNRs‐4′ demonstrated narrow size distribution. Transmission electron microscopy (TEM) images of CTAB‐AuNRs‐4′ (supernatant AuNRs fractions) confirmed the morphology uniformity with the particle length and width of 47.8±3.1 and 12.8±1.5 nm, respectively (Figure 2C). CTAB‐AuNRs‐4′ (supernatant AuNRs fractions) occupied 18.3‐40.8% of the total Au amount determined by inductively coupled plasma mass spectrometry (ICP‐MS). Overall, the two‐step purification method did not only remove excess CTAB from the suspension, but it unexpectedly caused further LLSPR peak redshift and refinement of shape purity so that an ultrapure AuNRs sample from the supernatant fraction of LLSPR peak and L/T ratio of 848 nm and 5.49, respectively, was produced.

### 2.3 CTAB‐AuNRs are Colloidally and Optically Stable in Deionized Water

**Figure 3:**
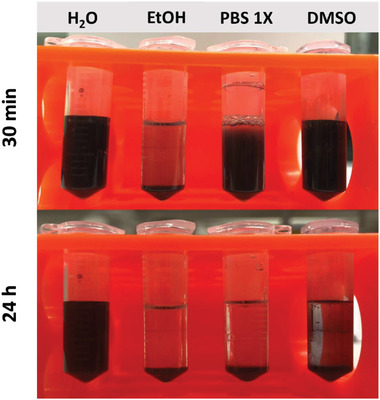
Colloidal stability of CTAB‐AuNRs in different solvents at 30 min and 24 h. The purified CTAB‐AuNRs were divided into four fractions and mixed with 1 mL of deionized water (H_2_O), ethanol (EtOH), PBS (1×, pH 7.4), and dimethyl sulfoxide (DMSO). Photomicrographs were taken at the specified timepoints.

CTAB‐AuNRs demonstrated the highest colloidal stability in deionized water followed by dimethyl sulfoxide (DMSO) > phosphate‐buffered saline (PBS) 1X > ethanol over the 24 h period studied (**Figure** **3**). After 4 days storage of CTAB‐AuNRs suspension in deionized water at RT, a redshift from 858 nm to 862 nm without broadening or tailing of the peak was demonstrated (Figure S4, Supporting Information) suggesting their stability. AuNRs were stored in deionized water until further use.

### 2.4 Physically and Optically Stable PEG‐AuNRs were Successfully Synthesized

**Scheme 1:**
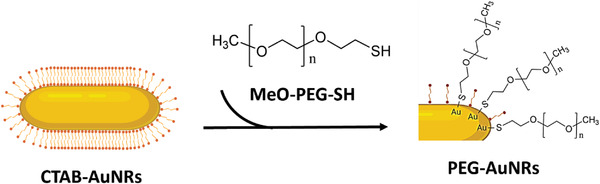
CTAB exchange with PEG for formation of PEG‐AuNRs. The thiol group in MeO‐PEG‐SH can conjugate with gold to form the Au‐S bond.

**Figure 4:**
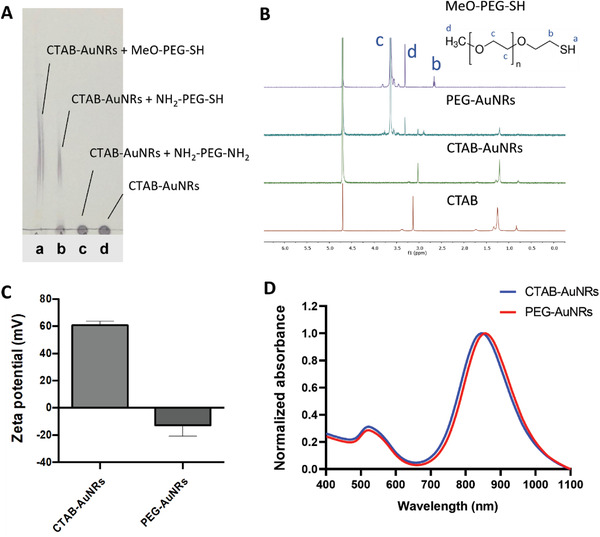
Physico‐chemical characterization of PEG‐AuNRs and CTAB‐AuNRs. A) Products after CTAB‐AuNRs reaction with a) MeO‐PEG‐SH, b) NH_2_‐PEG‐SH, and c) NH_2_‐PEG‐NH_2_. d) CTAB‐AuNRs starting material was used as a control. Developing solvent: chloroform‐methanol mixture (1/1, v/v) with 10% (v/v) ammonium solution. B) ^1^H NMR spectra (400 MHz) of MeO‐PEG‐SH, PEG‐AuNRs, CTAB‐AuNRs, and CTAB using D_2_O as solvents. The assigned peaks of panels (b–d) represent the corresponding parts of MeO‐PEG‐SH. In ^1^H NMR, the ‐SH peak was absent due to the proton exchange with D_2_O. C) Zeta potential of CTAB‐AuNRs and PEG‐AuNRs. D) Normalized UV–vis‐NIR spectra of PEG‐AuNRs and CTAB‐AuNRs.

In order to apply AuNRs for biological applications, PEG is a favorable stabilizing agent to use compared to CTAB. In this work, we have used CTAB‐AuNRs as a precursor to produce PEG‐AuNRs, as shown in **Scheme** **1**. Conjugation was confirmed by several methods. Retention factors (Rf) value of PEG obtained by thin layer chromatography (TLC) was ≈0.9 under developing with a mixture of chloroform and methanol (1/1, v/v) with 10% (v/v) ammonia solution as a mobile phase (Figure S5, Supporting Information). AuNRs modified with MeO‐PEG‐SH or NH_2_‐PEG‐SH migrated on the TLC plate (**Figure** **4**A(a,b)) while the negative reaction control, AuNRs mixed with NH_2_‐PEG‐NH_2_ and CTAB‐AuNRs, remained at the application point (Figure 4A(c,d)). ^1^H NMR spectra of PEG‐AuNRs presented a typical sharp peak of MeO‐PEG‐SH (‐OC *H_2_*‐C *H*_2_O‐) repeat units at 3.6 ppm (Figure 4B). CTAB traces in PEG‐AuNRs were detected suggesting that CTAB could not be completely removed.

After PEG modification, the size and morphology of AuNRs were maintained (Figure S6, Supporting Information). The zeta potential of PEG‐AuNRs however decreased dramatically to −12.7 ± 8.1 mV from 60.9 ± 2.8 mV (Figure 4C) suggesting successful conjugation of PEG. The LLSPR peak of PEG‐AuNRs demonstrated a slight redshift from 845 to 856 nm (Figure 4D), which may have occurred due to change in the dielectric constant around the AuNRs by the replacement of the ligands. No significant broadening or tailing of the longitudinal peaks was observed in UV‐vis‐NIR spectra, indicating the absence of aggregates formation.

The results altogether suggested successful functionalization of AuNRs with PEG and proposed that this is likely to have occurred through Au‐S coordination and is not a result of non‐specific binding to PEG.

### 2.5 Cytotoxicity Study

It is widely reported that PEG modification offers an improved biocompatibility profile compared to CTAB‐AuNRs.^[^ ^14^ ^]^ When evaluating the cytotoxicity of the particles based on a colorimetric reaction, the interference of the gold nanoparticles must be taken into consideration. We first attempt to evaluate the cytotoxicity of AuNRs using MTT assay. The solubilized formazan produced by viable cells read at 570 nm interferes with AuNRs absorbance window making MTT assay redundant to use which was confirmed by the false positive results obtained in our study at high rod concentration (Figure S7, Supporting Information). The modified MTT assay and the modified lactate dehydrogenase (LDH) assay were then applied which served the purpose since AuNRs were eliminated from the cell lysates by centrifugation (4000 rpm for 1 h, RT) before absorbance measurement.

**Figure 5:**
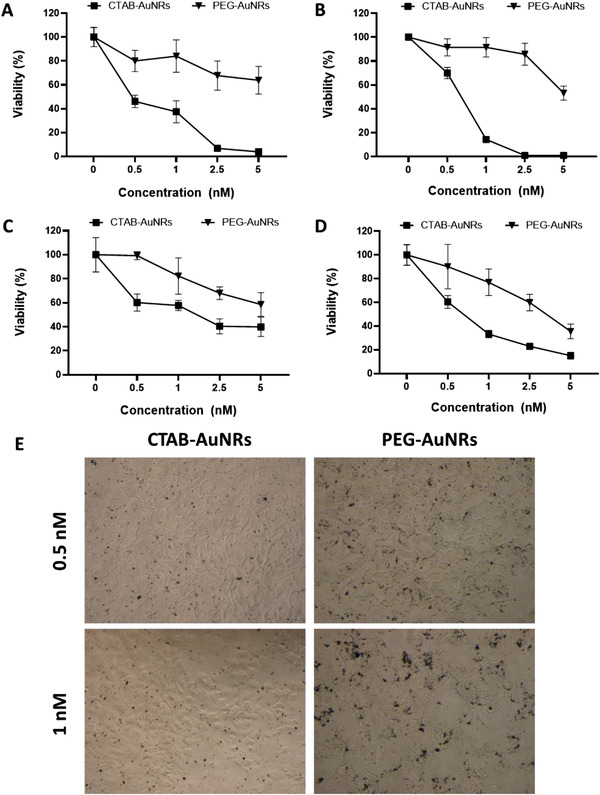
Cytotoxicity of CTAB‐AuNRs and PEG‐AuNRs. Cytotoxicity of CTAB‐AuNRs and PEG‐AuNRs in SN4741 (A,C) and B16F10 (B,D) cells measured by the modified LDH assay (A,B) and the modified MTT assay (C,D), respectively. Cells were seeded in 96‐well plates at 6×10^3^ cells/well seeding density and then treated with the particles in the concentration over 0.5 and 5 nm concentration range for 24 h. Cell viability was expressed as mean ± SD, *n* = 3–5. E) Light microscopy images (200× magnification) of CTAB‐AuNRs and PEG‐AuNRs after 24 h treatment.

LDH assay determines the released LDH upon damage to the cell membrane while MTT assay quantifies mitochondrial activity by measuring the formazan formed by the reduction of the MTT. Both AuNRs derivatives exhibited dose‐dependent toxicities. Expectedly, CTAB‐AuNRs exhibited more pronounced toxicity than PEG‐AuNRs in both SN4741 cells and B16F10 cells confirmed by the modified LDH assay and the modified MTT assay (**Figure** **5**A–D). In case of CTAB‐AuNRs, the modified LDH assay appeared more sensitive than the modified MTT assay presumably due to the membrane permeabilising nature of CTAB. MeO‐PEG‐SH was innocuous to cells at the experimental concentrations, while almost all cells were dead when the concentration of CTAB was higher than 0.1 mm (Figure S8, Supporting Information). Light microscopy images (Figure 5E) confirmed the ability of PEG‐AuNRs to interact with cells suggesting that lack of toxicity is not due to uptake inhibition but is genuinely due to improved biocompatibility profile of AuNRs constructs following PEGylated and reducing CTAB content.

## 3 Discussion

A typical seed‐mediated growth method includes two steps: the seed nanoparticle synthesis and subsequent seed growth using solutions containing metal precursors, reducing reagents, and shape‐directing reagents.^[^ ^15^ ^]^ One popular mechanism proposed for AuNR synthesis *via* seed‐mediated growth method is that small gold seeds are formed first through rapid reduction by NaBH_4_ which serves as nucleation sites for subsequent growth of gold nanorods.^[^ ^16^ ^]^ In the growth system, when using vitamin C as a weak reducing agent, AuCl_4_^−^ are reduced to AuCl_2_^−^. In the presence of the gold seeds, these AuCl_2_^−^ ions can be further reduced to gold atoms. With the help of surfactants and silver ions, breaking the symmetry by preferential deposition on the {110} of gold lateral facets, the growth of end surfaces is boosted so anisotropic gold nanorods are formed.^[^ ^12^ ^]^ Our results support this working hypothesis as the more AgNO_3_ added, the larger of LLSPR peak wavelength of AuNRs was generated in line with published work.^[^ ^17^, ^18^, ^19^ ^]^ Under the AuNRs growth conditions reported in this study, it is unlikely that Ag^+^ is reduced to bulk silver atoms. It is however possible that underpotential deposition (UPD) occurs. In this case, Ag^+^ is reduced to Ag^0^ which forms a metal monolayer onto the metal substrate at a much lower potential compared to reduction in the bulk media. Orendorff and Murphy proposed the silver UPD mechanism to elucidate the silver‐assisted nanorod growth process.^[^ ^20^, ^21^ ^]^ When adding the seed solution to the growth solution, AuCl_2_^−^ on the CTAB micelles diffuses, by electric field interactions, to the CTAB‐capped seed spheres and break the sphere symmetry into different facets. Silver ions deposit faster onto the {110} side facet than the end facet, resulting in rod shape particle growth. The effect of the silver ion content is not always to increase the LLSPR of AuNRs, an increase in silver ions over a threshold concentration, due to the blocking of the end facet of the nanorods by silver metal deposition, can no longer increase AuNRs longitudinal growth. Feng et al. reported that excessive amounts of AgNO_3_ in the growth solution resulted in gold nanospheres formation.^[^ ^17^ ^]^ El‐Sayed et al. reported that a reduction in LLSPR of the AuNRs when excessive AgNO_3_ was used.^[^ ^12^ ^]^ In our study, when > 2.25 mL AgNO_3_ was used, multiple absorbance peaks were observed in the UV‐vis‐NIR spectra suggesting formation of heterogeneous particles. This effect was counteracted by addition of a small amount of HCl which slowed down gold ions reduction rate leading for formation of CTAB‐AuNRs with a single and dominant LLSPR peak with the wavelength > 800 nm.

Although AuNRs can be generated using the seed‐mediated growth method, shape impurity remains a major challenge for AuNRs synthesis.^[^ ^22^ ^]^ Slight variations in reactant concentrations, reaction temperature and reagent aging during synthesis all can result in particle morphology, optical properties batch‐to‐batch variations.^[^ ^8^, ^10^, ^22^ ^]^ It is reported that CTAB from different suppliers or different catalogue numbers not only can affect the aspect ratio of the nanorods, but also the yield due to the existence of impurities.^[^ ^20^ ^]^

Spherical particles are considered the main by‐products of the seed‐mediated growth method which have strong absorbance at 520 nm causing an apparent increase in TLSPR peak. The L/T ratio, the intensity ratio of LLSPR peak to TLSPR peak, is an important and facile indicator of the rod shape purity. The higher L/T ratio the higher the rod shape purity is. Unlike conventional purification method where particles are collected anonymously by centrifugation (Method‐1), the modified two‐step purification method employed in this study (Method‐2) resulted in L/T ratio that is five times higher for AuNR collected from the supernatant (CTAB‐AuNRs‐4′, L/T = 5.49, 18.3‐40.8% w/w yield) compared to those retained in the pellets (CTAB‐AuNRs‐3′, L/T = 1.05, 59.2‐81.7% w/w yield). The dialysis step was crucial as the removal of excess CTAB reduced the overall dispersibility of the colloidal particles making the effect of shape‐related drag coefficient on particles sedimentation rates more apparent. Xiong et al. applied density gradient sucrose centrifugation to purify a heterogeneous nanorod colloid mixture and reported the following sedimentation rates: large spheres > short and fat rods > long and thin rods > light spheres and rods.^[^ ^23^ ^]^ We applied centrifugation to separate rods from spherical and cubic particles after dialysis. We hypothesized that removal of excess CTAB by dialysis will reduce the overall dispersibility of the particles allowing shape and density effects to dominate when subjected to centrifugation. The dialysis/centrifugation combinatorial purification approach allowed rod‐shaped particles to remain suspended, i.e., in the supernatant fraction, while heavy spherical/cubical particles pelleted more easily when centrifugation was applied. TEM, redshifted LLSPR peak by 848 nm, and an increased L/T ratio of 5.49 for the supernatant fractions are supportive of this hypothesis. This facile purification protocol can be useful particularly when batch‐to‐batch synthesis variations are encountered.

After synthesis, CTAB is firmly absorbed on the AuNRs surface, forming a dense surface‐confined cationic bilayer with the trimethylammonium head towards the external environment.^[^ ^24^ ^]^ This cationic bilayer can generate defects in the cell membrane and induce cell death.^[^ ^25^ ^]^ Developing a surface functionalization strategy to replace CTAB with more biocompatible ligands without causing particle aggregation is required.

The direct interaction of the gold surface with thiols (−SH) and disulfides (−S−S−), forming stable Au‐S bonds, is the most widely applied approach for grafting a coating on gold nanoparticles.^[^ ^26^ ^]^ It was proposed that a coordinate bond between the –SH group and gold surface is formed first. Followed by the dissociation of S–H bond which results in a thiyl radical, a gold‐thiolate covalent bond at the gold‐sulfur interface is formed eventually.^[^ ^27^ ^]^ The bond energy is ≈40 kcal mol^−1^.^[^ ^24^ ^]^ The stability of the Au‐S bond is compromised by the increased temperature, e.g., from lower than 40 to 42 °C, and the change of the chemical composition of the solvent, e.g., from water to PBS and culture media solutions.^[^ ^26^ ^]^ Thiol containing compounds with various chemical structures have different affinities to gold nanoparticles. Gold nanoparticles modified with PEG‐SH demonstrated the highest stability in aqueous solution compared with other thiol compounds including glutathione, mercaptopropionic acid, cysteine, cystamine, and dihydrolipoic acid.^[^ ^28^ ^]^ In this work, AuNRs have been used at physiological temperature hence no temperature stability studies were carried out. Future studies involving hyperthermia should include stability assessment in their design.

## 4 Conclusions

In this study, we report an optimized synthesis protocol to produce CTAB‐AuNRs with an LLSPR value of > 800 nm falling in the biological transparency window. We also report a two‐step purification method, dialysis followed by centrifugation, for efficient removal of CTAB, further redshifting LLSPR peak and increasing shape purity of the rods. The purified AuNRs were successfully PEG‐functionalized rendering them more biocompatible and suitable for biological applications.

## 5 Experimental Section

### Material and Reagents

Gold(III) chloride trihydrate (HAuCl_4_ · 3H_2_O, ≥99.9%), cetyltrimethylammonium bromide (CTAB, ≥99%), sodium borohydride (NaBH_4_, ≥96%), l‐ascorbic acid (Vitamin C, ≥99%), O‐(2‐Mercaptoethyl)‐O′‐methylpolyethylene glycol (MeO‐PEG‐SH, average Mw = 2000), and thiazolyl blue tetrazolium bromide (MTT) were purchased from Sigma–Aldrich (UK). Hydrochloric acid (HCl, 36.5‐38.0%) was purchased from Honeywell (UK). Amine‐PEG‐amine (NH_2_‐PEG‐NH_2_, average Mw = 3500) and amine‐PEG‐thiol (NH_2_‐PEG‐SH, average Mw = 3500) were purchased from JenKem Technology (USA). Silver nitrate (AgNO_3_, ≥99.8%) was supplied by VWR International (UK). Fetal calf serum (FCS), penicillin‐streptomycin, GlutaMAX^TM^, 0.5% trypsin‐EDTA, PBS (pH 7.4, 10×), and 16% (w/v) formaldehyde were obtained from Thermo Fisher Scientific (UK). CytoTox 96® non‐radioactive cytotoxicity assay kit for the modified LDH assay was purchased from Promega (USA). Ultra‐pure water was produced by a PURELAB® water purification system (USA) with a resistivity of 18.2 MΩ cm. Dialysis bags (SnakeSkin Dialysis Tubing, MWCO = 3500) were purchased from Thermo Fisher Scientific (UK). Other chemicals, not specified here, were all analytical grades purchased from standard chemical suppliers and used without further purification.

### Synthesis of CTAB‐AuNRs

AuNRs were synthesized by the published seed‐mediated growth method with some modifications.^[^ ^11^, ^12^, ^13^ ^]^ The seed solution was prepared as follows: CTAB solution (5 mL, 0.2 m) was mixed with HAuCl_4_ · 3H_2_O (5 mL, 0.0005 m). Then ice‐cold NaBH_4_ (600 µL, 0.01 m) was added into the above mixture forming a brownish‐yellow solution. The solution was vigorously stirred for 2 min and kept in a water bath at 28 °C for at least 2 h before use.

For growth solution preparation, HAuCl_4_ · 3H_2_O (45 mL, 0.001 m) was mixed with AgNO_3_ (0.90, 2.25, or 3.60 mL, 0.004 m) and CTAB (45 mL, 0.1 m). Solutions were mixed a few times by inversion, followed by addition of Vitamin C (0.72 mL, 0.1 m), at which point the color of the growth solution changed from dark yellow to colorless. HCl solution (0, 0.36, 0.72, 1.08, or 1.44 mL, 1 m) was then added. To form CTAB‐AuNRs, the seed solution (90, 180, or 360 µL) was added into the growth solution prepared above and was kept at 28 °C overnight.

### Purification of CTAB‐AuNRs

After synthesis, the mixtures were purified by one of two methods to remove excess CTAB and collect CTAB‐AuNRs.

**Method‐1**: The sample was purified by centrifugation only. After synthesis, AuNRs suspension was divided equally into two 50 mL centrifuge tubes and centrifuged (Centrifuge 5810 R, Eppendorf, UK) at 10 000 rpm for 30 min at room temperature (RT). The pellet fraction (containing particles of all shapes) was collected, re‐dispersed in 20 mL deionized water, and washed again by centrifugation at 10 000 rpm, 30 min, RT before use.

**Method‐2**: AuNRs suspension was purified by a combinatory purification method. First, an overnight dialysis against 1500 mL deionized water, changed 2–3 times maintaining sink conditions was applied to reduce CTAB concentration. The dialyzed AuNRs suspension was equally divided into two 50 mL centrifuge tubes and centrifuged at 10 000 rpm, 15 min, RT. The pellet (containing particles of all shapes) or the supernatant (containing AuNRs with high morphology uniformity) were collected and stored at RT before use.

### Colloidal Stability of CTAB‐AuNRs in Different Solvents

The collected CTAB‐AuNRs were dispersed into 1 mL of deionized water, ethanol, PBS (1×, pH 7.4), or DMSO. The colloidal stability of CTAB‐AuNRs in different solvents was evaluated at 30 min and 24 h.

### Preparation of PEG‐AuNRs

PEG‐modified AuNRs (PEG‐AuNRs) were prepared as previously reported with some modifications.^[^ ^29^ ^]^ In brief, MeO‐PEG‐SH (200 µL, 5 mm), or NH_2_‐PEG‐SH (200 µL, 5 mm) and NH_2_‐PEG‐NH_2_ (200 µL, 5 mm) as reaction controls, was added to CTAB‐AuNRs (1 mL, 50 nm). The mixture was stirred for 24 h at RT. At the end of the reaction, the mixtures were centrifuged (10 000 rpm, 15 min, RT) (Fresco 21 Microcentrifuge, Thermo Fisher Scientific, UK). The pellet was redispersed in deionized water (1 mL) and centrifugated at 10 000 rpm, 15 min, RT to wash once before use.

### UV–Vis–NIR Absorption

The optical properties of purified CTAB‐AuNRs and PEG‐AuNRs were investigated by a UV/VIS spectrometer (Lambda2, Perkin Elmer, USA) with a wavelength range of 400–1100 nm. The intensity ratio of LLSPR peak to TLSPR peak (L/T) was calculated as a facile indicator to evaluate the rod purity. The AuNR fraction with the L/T ratio higher than three signifying AuNRs with high shape purity was collected.

### Zeta Potential

AuNRs were dispersed in deionized water in a disposable plain folded capillary Zeta cells. Zeta potential of the particles was determined by electrophoretic mobility measurement (Zetasizer Nano series, Malvern Instruments, UK) at RT.

### AuNRs Yield After Purification

Au amount in CTAB‐AuNR pellet and supernatant fractions purified by Method‐2 was quantified by ICP‐MS (n = 5) (PerkinElmer, USA). First, 10 µL of AuNR was dissolved in 990 µL of aqua regia and digested in an oven at 60 °C overnight. Samples were then diluted in ultra‐pure water to 10 mL before measurement. ICP‐MS operating conditions: RF power, 1600 W; Ar Gas flow, 1 L min^−1^; Ar Auxiliary gas flow, 1.2 L min^−1^; Ar Plasma flow, 18 L min^−1^; Nebulizer gas flow, 0.94 L min^−1^; mode of operation, standard. Au content was determined using a 0.02–5000 µg L^−1^ calibration curve (*R* ^2^ = 1) of the standards.

### Surface Functionalization with PEG Thiol

Successful PEG modification was confirmed by TLC using a mixture of chloroform and methanol (1/1, v/v) with 10% (v/v) ammonia solution as a developing solvent and proton nuclear magnetic resonance (^1^H NMR, 400 MHz) spectrometer (Ascend^TM^ 400, Bruker, UK) using D_2_O as a solvent.

### AuNR Particle Size and Molar Concentration Determination

Size distribution and molar concentration of AuNRs were determined by NTA (NanoSight LM10, Malvern Instruments, UK). Measurements were done by diluting the stock solution in filtered deionized water to obtain 20–80 particles in the viewing frame. The modal size and particle count were measured in quadruplicate, with 30 s as the duration for each recording, and analyzed using the NanoSight NTA 3.2 software (Malvern Instruments, UK).

### Morphology

The morphology of CTAB‐AuNRs and PEG‐AuNRs was evaluated by TEM. A drop of the stock suspension was deposited onto carbon‐coated 300‐mesh copper grids and dried in air. The grid was quickly plotted with a filter paper and rinsed once with deionized water before imaging with a Philips CM 12 transmission electron microscope (FEI Electron Optics, The Netherlands) equipped with Tungsten filament and a Veleta – 2k × 2k side‐mounted TEM CCD camera (Olympus, Japan) at the accelerating voltage of 80 kV.

### Cell Culture

SN4741 cells, a mouse midbrain neuronal cell line, were cultured using DMEM (high glucose) supplemented with 10% heat‐inactivated fetal calf serum, 1% penicillin‐streptomycin, 1% GlutaMAX^TM^, and 0.6% glucose. B16F10 cells, a mouse melanoma cell line, were cultured using RPMI‐1640 medium supplemented with 10% fetal calf serum, 1% penicillin‐streptomycin and 1% GlutaMAX^TM^. Cells were maintained at 37 °C under a humidified atmosphere containing 5% CO_2_ and were subcultured using trypsin‐EDTA at 70–80% confluency.

### Cell Viability

Cytotoxicity of CTAB‐AuNRs and PEG‐AuNRs were measured in SN4741 cells and B16F10 cells, respectively. Cells were seeded in 96‐well plates at a density of 6 × 10^3^ cells per well, left to adhere overnight then treated with 100 µL of CTAB‐AuNRs or PEG‐AuNRs at a range of 0.5–5 nm. MeO‐PEG‐SH at the experimental concentrations and CTAB were used as controls. Modified LDH assay and modified MTT assay were used to investigate the cytotoxicity of the particles.

### Modified LDH Assay

Cell viability was evaluated using the modified LDH assay following published protocols with some modifications.^[^ ^30^ ^]^ Lysis solution was prepared by diluting 9% Triton X‐100 with phenol‐free medium to a final of 0.9% Triton X‐100 concentration. After 24 h incubation, the medium was removed. Cells were washed with PBS (1×, pH 7.4) once and a 100 µL of lysis solution was added for 1 h before centrifugation at 4000 rpm for 1 h at RT. Afterward, 30 µL of AuNR‐free supernatant (or the lysis solution as the negative control) were mixed with 30 µL of LDH reagent in a 96‐well plate, followed by 20 min incubation in the dark, then quenched by adding 30 µL of stop solution to each well. Absorbance was measured at 490 nm in an FLUO star OPTIMA plate reader (BMG Labtech, USA). Results were expressed as the percentage cell viability and calculated using the following equation:

(1) $$\begin{array}{ccc} & & \mathrm{Cell}\;\mathrm{viability}\;\left(\%\right)\\ & & \;=\frac{A490\;\mathrm{nm}\;\mathrm{of}\;\mathrm{treated}\;\mathrm{cells}-A490\;\mathrm{of}\;\mathrm{negative}\;\mathrm{control}}{A490\;\mathrm{nm}\;\mathrm{of}\;\mathrm{untreated}\;\mathrm{control}\;\mathrm{cells}-A490\;\mathrm{of}\;\mathrm{negative}\;\mathrm{control}}\times 100 \end{array}$$

### Modified MTT Assay

At the end of the incubation period, the media was removed and replaced with 120 µL of MTT solution at a final concentration of 0.5 mg mL^−1^. Cells were incubated for 4 h at 37 °C and 5% CO_2_. The formazan was dissolved in 200 µL of DMSO. The plate was centrifuged at 4000 rpm for 1 h at RT to eliminate interference from the cell‐associated AuNRs. The supernatant was transferred in a 96‐well plate and read at 570 nm in the plate reader. Results were expressed as the percentage cell viability and calculated using the following equation:

(2) $$\mathrm{Cell}\;\mathrm{viability}\;\left(\%\right)\;=\;\frac{A570\;\mathrm{nm}\;\mathrm{of}\;\mathrm{treated}\;\mathrm{cells}}{A570\;\mathrm{nm}\;\mathrm{of}\;\mathrm{untreated}\;\mathrm{control}\;\mathrm{cells}}\times 100$$

### Cell Morphology

Light microscopy images were captured using a Leica microscope with Q‐capture pro 7 software after 24 h treatment with CTAB‐AuNRs or PEG‐AuNRs at the concentration of 0.5 or 1 nm. B16F10 cells were washed twice with PBS (pH 7.4) and fixed with 4% (v/v) formaldehyde before imaging.

### Data Analysis

Quantitative results were presented as mean ± standard deviation (SD), where “n” denotes the number of repeats.

## Conflict of Interest

The authors declare no conflict of interest.

## Supporting information

Supporting Information

## Data Availability

The data that support the findings of this study are available from the corresponding author upon reasonable request.
