# Supplementary material for: Combined Facile Synthesis, Purification, and Surface Functionalization Approach Yields Monodispersed Gold Nanorods for Drug Delivery Applications
Source: Part Part Syst Charact. 2023 Sep 5;40(10):2300043. doi: 10.1002/ppsc.202300043 (PMC10777591; doi:10.1002/ppsc.202300043)
Supplement: Supplementary file 1 — Supporting Information [file PPSC-40-2300043-s001.pdf]

Particle
& Particle Systems Characterization

Supporting Information

for *Part. Part. Syst. Charact.*, DOI 10.1002/ppsc.202300043

Combined Facile Synthesis, Purification, and Surface Functionalization Approach Yields Monodispersed Gold Nanorods for Drug Delivery Applications

*Shunping Han and Khuloud T. Al-Jamal**

Supplementary Information

Combined facile synthesis, purification and surface functionalization approach yields monodispersed gold nanorods for drug delivery applications

*Shunping Han and Khuloud T. Al-Jamal**

S. Han, K. T. Al-Jamal
Institute of Pharmaceutical Science, Faculty of Life Sciences & Medicine, King's College London, Franklin-Wilkins Building, 150 Stamford Street, London SE1 9NH, United Kingdom
E-mail: khuloud.al-jamal@kcl.ac.uk

Keywords: gold nanorods, optimization, synthesis, purification, cytotoxicity

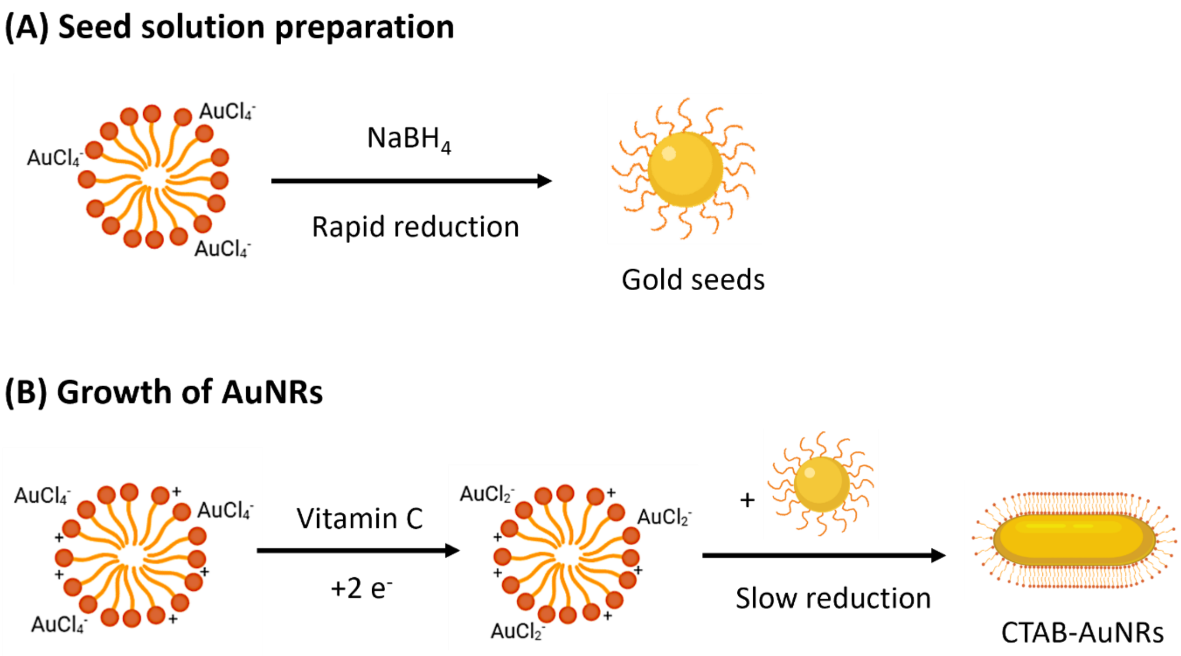

Figure S1. Chemical mechanism of CTAB-AuNRs synthesis. (A) Gold seeds are formed first through rapid reduction by $NaBH_4$ and then added as nucleation centres. **(B)** In the growth procedure, $AuCl_4^-$ ions are bound to the cationic CTAB micelles and then reduced to $AuCl_2^-$ by using vitamin C as a weak reducing agent. Only with the presence of the gold seeds, $AuCl_2^-$ ions can be further reduced to gold atoms. This deposition particularly prefers to occur at the tips of the seeds, ultimately generating rod formation. *This figure was adapted from Pérez-Juste, et al.2004 with minor modifications.* [1]

Table S1. Optimization of seed solution, $AgNO_3$ solution and HCl solution volumes for the synthesis of CTAB-AuNRs with the longest LLSPR.

Group No.	Seed Solution (μL)	Growth Solution (mL)					TLSPR (nm)	LLSPR (nm)
		$AgNO_3$ 0.004 M	HCl 1 M	$HAuCl_4$ 0.001 M	CTAB 0.1 M	Vitamin C 0.1 M		
1a	90						536	658
1b	180	2.25	/	45	45	0.72	544	622
1c	360						527	696
2a		0.90					522	704
2b	360	2.25	/	45	45	0.72	524	749, 835
2c		3.60					525	677, 935
3a			/				528	670
3b			0.36				522	738
3c	360	3.60	0.72	45	45	0.72	529	749
3d			1.08				526	792
3e			1.44				537	801

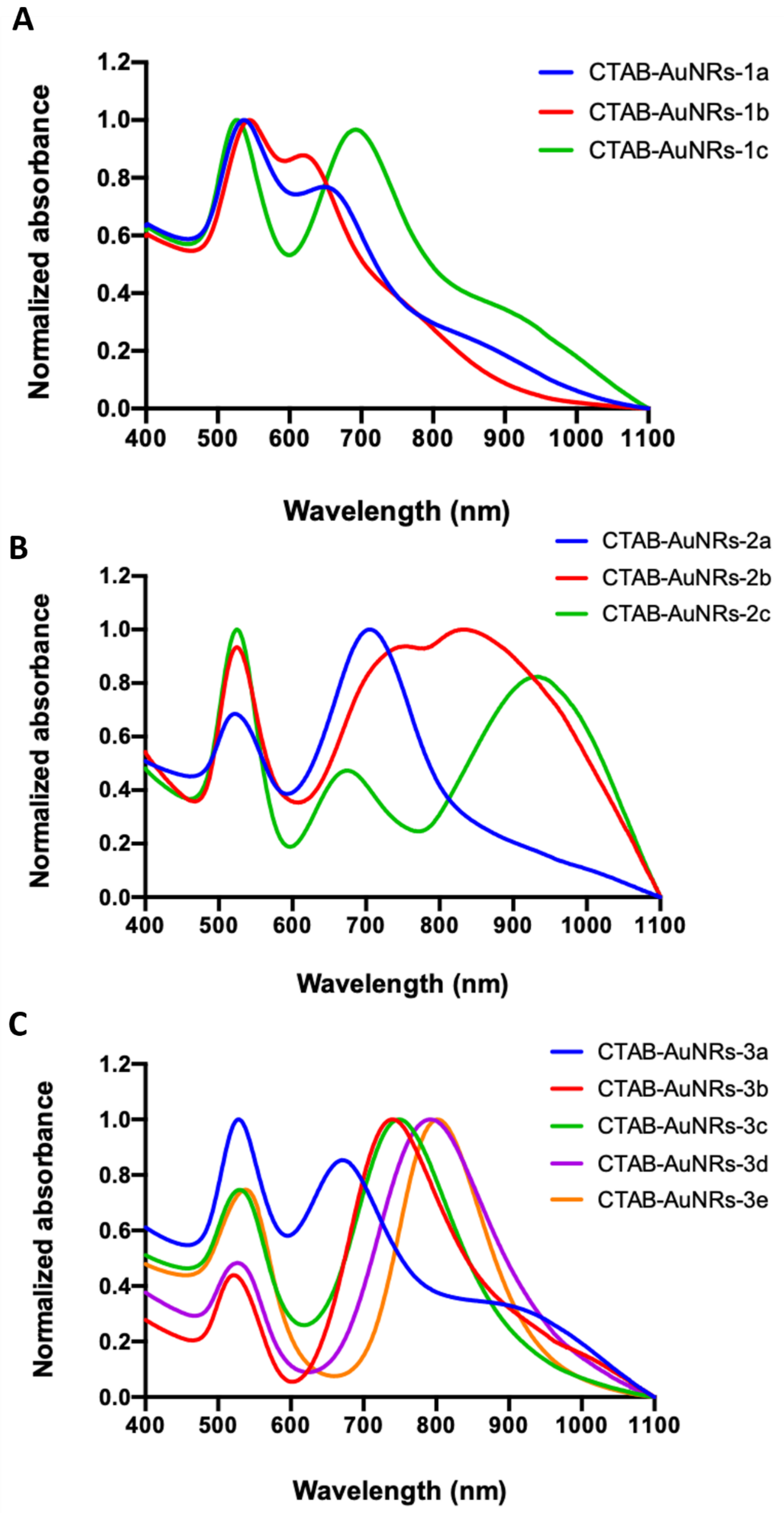
A
CTAB-AuNRs-1a
CTAB-AuNRs-1b
CTAB-AuNRs-1c
Normalized absorbance
Wavelength (nm)
B
CTAB-AuNRs-2a
CTAB-AuNRs-2b
CTAB-AuNRs-2c
Normalized absorbance
Wavelength (nm)
C
CTAB-AuNRs-3a
CTAB-AuNRs-3b
CTAB-AuNRs-3c
CTAB-AuNRs-3d
CTAB-AuNRs-3e
Normalized absorbance
Wavelength (nm)

Figure S2. Normalized UV-vis-NIR spectra of CTAB-AuNRs synthesized by varying the volumes of (A) seed solution, (B) $AgNO_3$ solution and (C) HCl solution to generate CTAB-AuNRs with longest LLSPR. Volumes are listed in table S1. Absorbance was measured over 400 -1100 nm wavelength range with a UV/VIS spectrometer. The optimized CTAB-AuNRs-3e demonstrated two dominant peaks with the longest LLSPR.

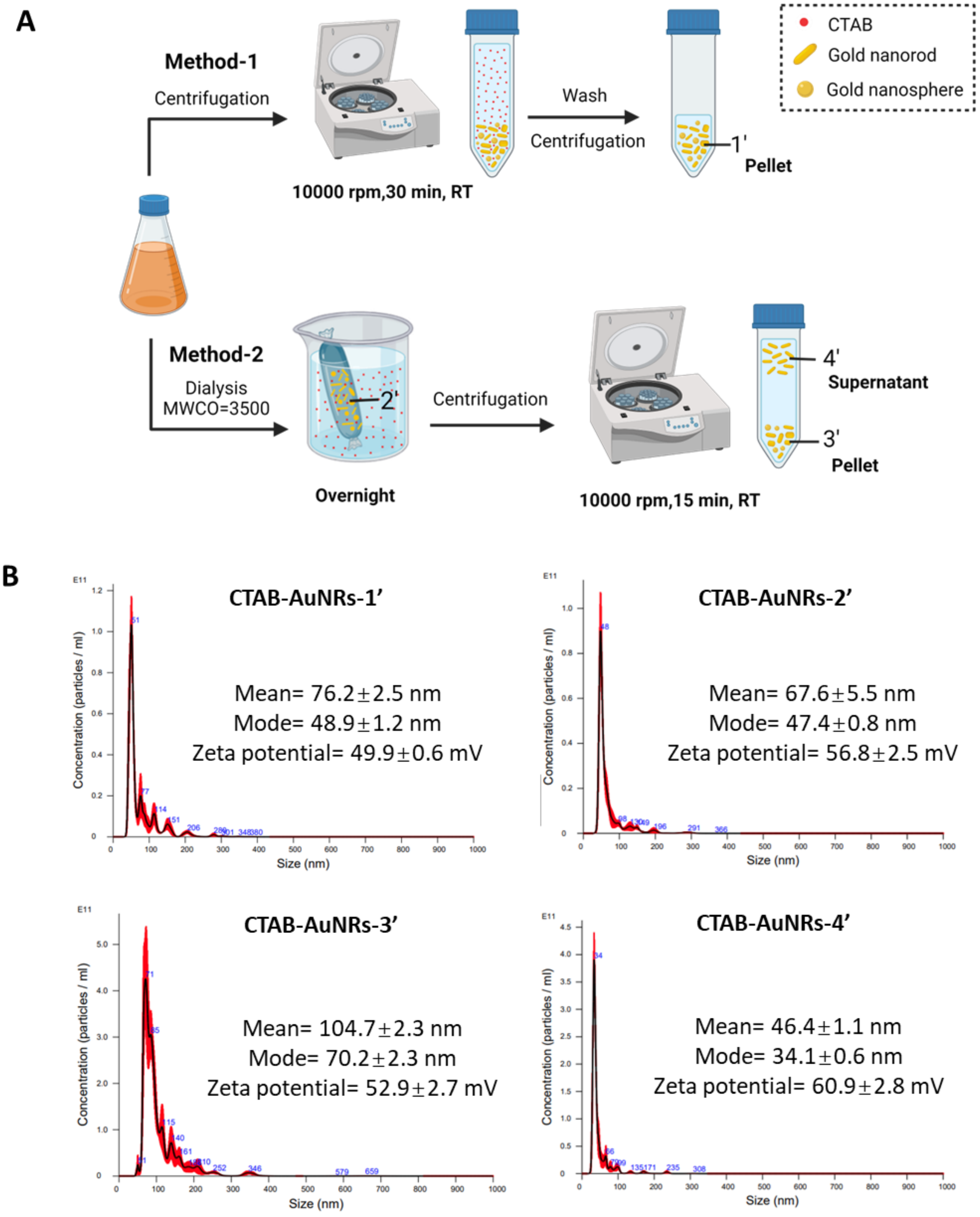

Figure S3. Hydrodynamic size distribution and zeta potential of CTAB-AuNRs in different fractions during purification. (A) Purification methods after CTAB-AuNRs synthesis. Method-1: sample was

purified by centrifugation (10,000 rpm, 30 min, RT). Method-2: sample was purified by a combinational method firstly by an overnight dialysis (MWCO=3500) followed by centrifugation (10,000 rpm, 15 min, RT). CTAB-AuNRs-1’: pellets obtained from method-1; CTAB-AuNRs-2’: suspension obtained after dialysis from method-2; CTAB-AuNRs-3’: pellets obtained from method-2; CTAB-AuNRs-4’: supernatant obtained from method-2 after centrifugation. **(B)** Hydrodynamic size distribution and zeta potential of CTAB-AuNRs in different fractions during purification measured by nanoparticle tracking analysis and Zetasizer Nano series, respectively.

Table S2. Hydrodynamic size distribution and zeta potential of CTAB-AuNRs in different fractions during purification.

**Compound**[1]	**Mean size**[2]	**D10**[3]	**D50**[3]	**D90**[3]	**Zeta potential**[4]
			(nm)		(mV)
CTAB-AuNRs-1’	76.2±2.5	44.1±1.3	56.6±3.5	134.4±8.5	49.9±0.6
CTAB-AuNRs-2’	67.6±5.5	43.3±1.0	54.2±3.1	101.6±13.4	56.8±2.5
CTAB-AuNRs-3’	104.7±2.3	66.7±2.2	86.0±2.0	173.8±9.8	52.9±2.7
CTAB-AuNRs-4’	46.4±1.1	29.1±0.7	35.3±0.3	70.4±3.3	60.9±2.8

[1] CTAB-AuNRs-1’: pellets obtained from method-1; CTAB-AuNRs-2’: suspension obtained after dialysis from method-2; CTAB-AuNRs-3’: pellets obtained from method-2; CTAB-AuNRs-4’: supernatant obtained from method-2 after centrifugation.
[2] Hydrodynamic size distribution was measured by nanoparticle tracking analysis (NTA).
[3] Percentile values. D10, D50 and D90 indicate the size below which 10%, 50% or 90% of all particles are found.
[4] Zeta potential was measured by electrophoretic mobility measurement using Zetasizer Nano series.

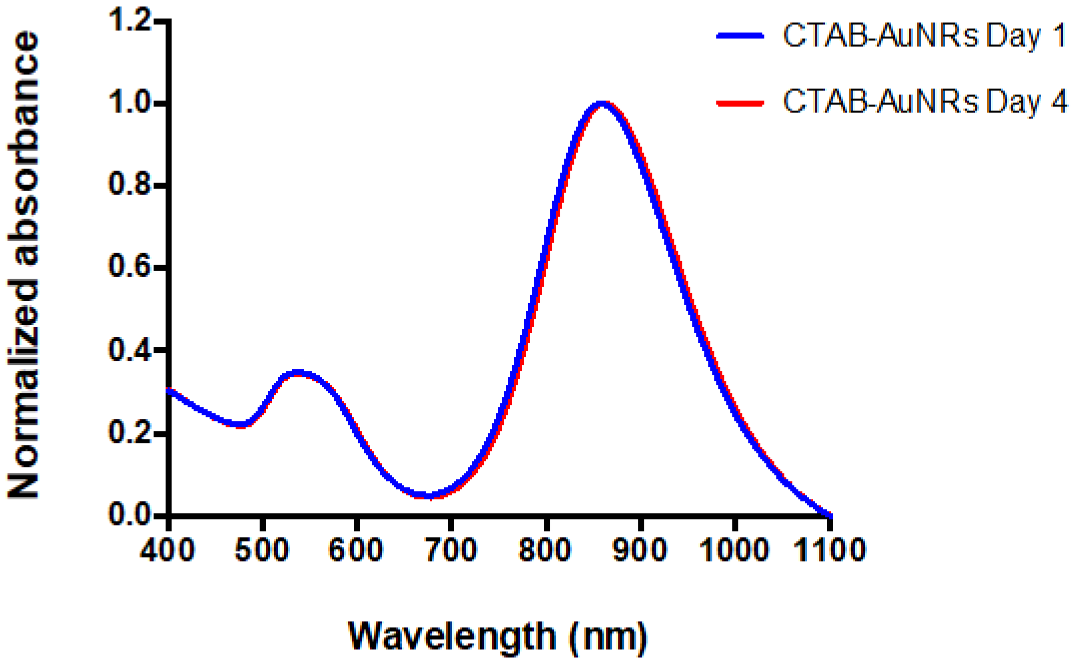

Figure S4. Normalized UV-vis-NIR spectra of CTAB-AuNRs at day 1 and day 4. Particles were dispersed in water. On day 4, the LLSPR of the CTAB-AuNRs slightly redshifted from 858 nm to 862 nm without broadening or tailing of the peak confirming their stability.

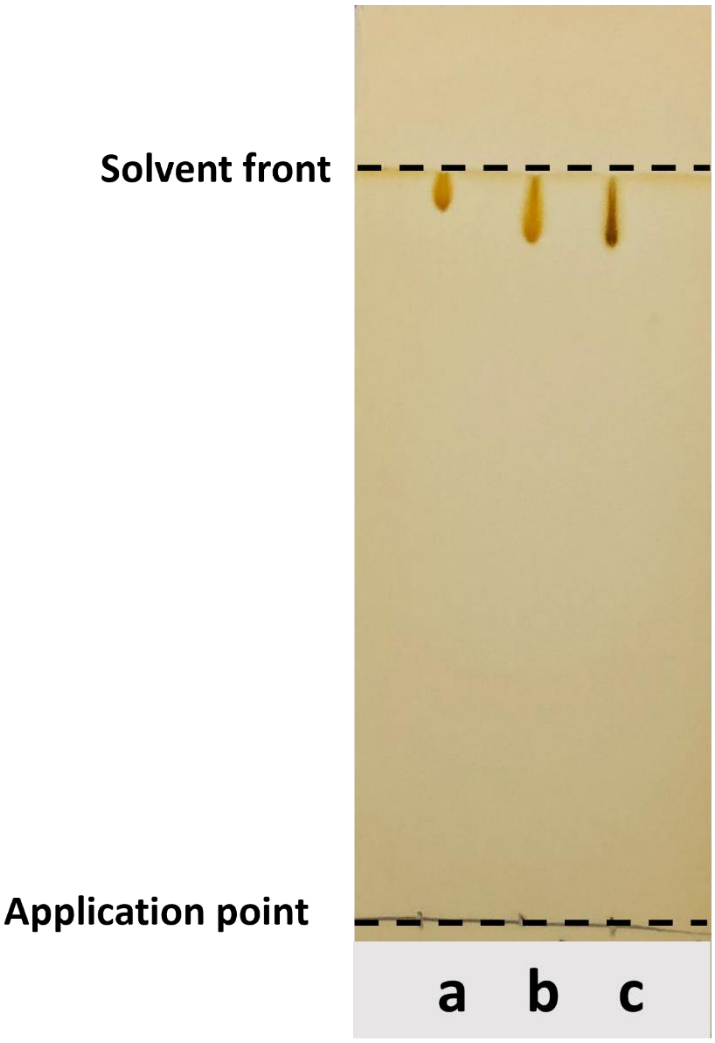

Figure S5. Migration of PEG in TLC plate. (a) MeO-PEG-SH, (b) NH_2-PEG-SH and (c) NH_2-PEG-NH_2. in TLC plate were developed using a mixture of chloroform and methanol (1/1, v/v) with 10% (v/v) ammonia solution as a mobile phase. The TLC plate was then dried in air and developed under iodine vapour for PEG detection.

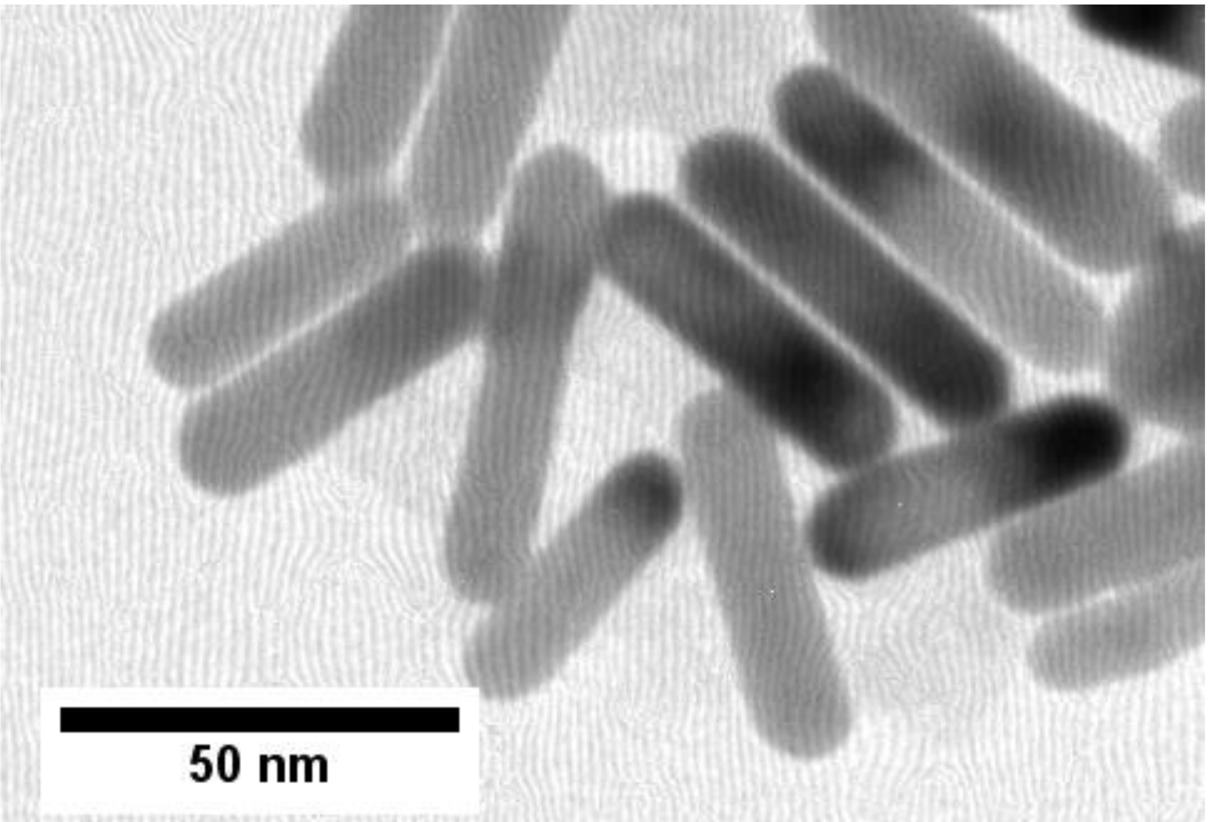

Figure S6. The TEM image of PEG-AuNRs. Sample was dropped onto carbon-coated 300-mesh copper grids, dried in air before imaging with a Philips CM 12 transmission electron microscope (FEI Electron Optics, The Netherlands) equipped with Tungsten filament and a Veleta - 2k × 2k side-mounted TEM CCD camera (Olympus, Japan) at the accelerating voltage of 80 kV. Rods appear well-dispersed despite CTAB removal. Scale bar =50 nm.

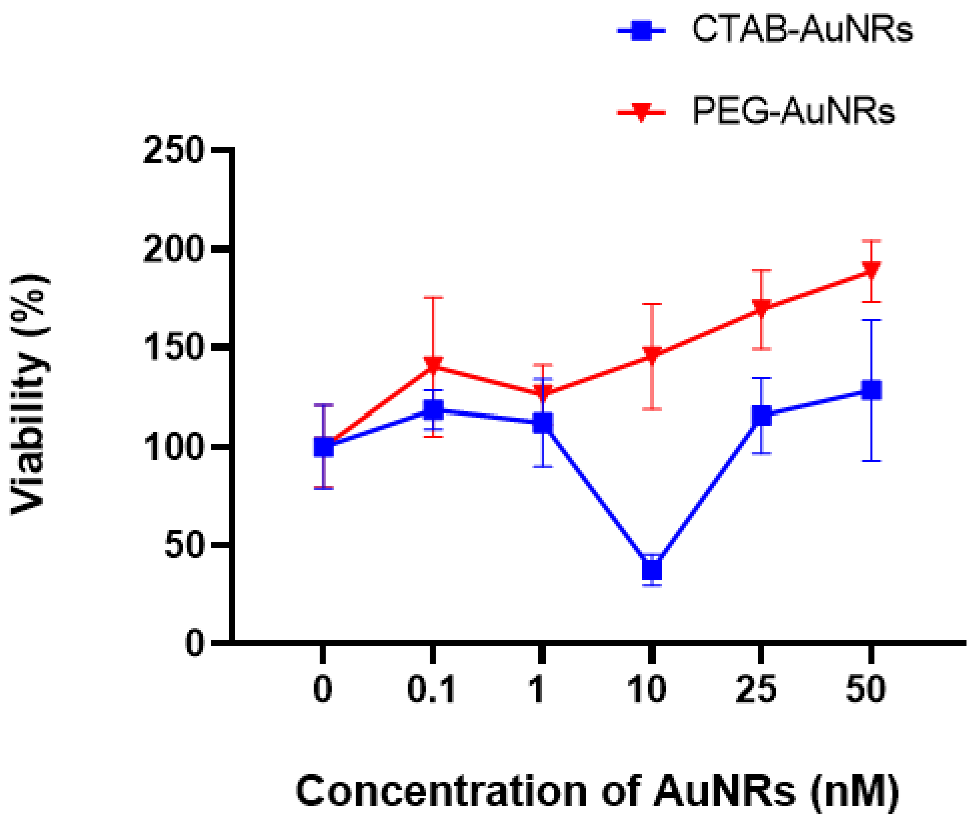

Figure S7. Cell viability of CTAB-AuNRs and PEG-AuNRs in B16F10 cells studied by MTT assay. Cells were seeded in 96-well plates at 6×10^3 cells/well and treated with the particles over 0.1 nM to 50 nM concentration range for 24 h. Cell viability was expressed as mean ± SD, n=3.

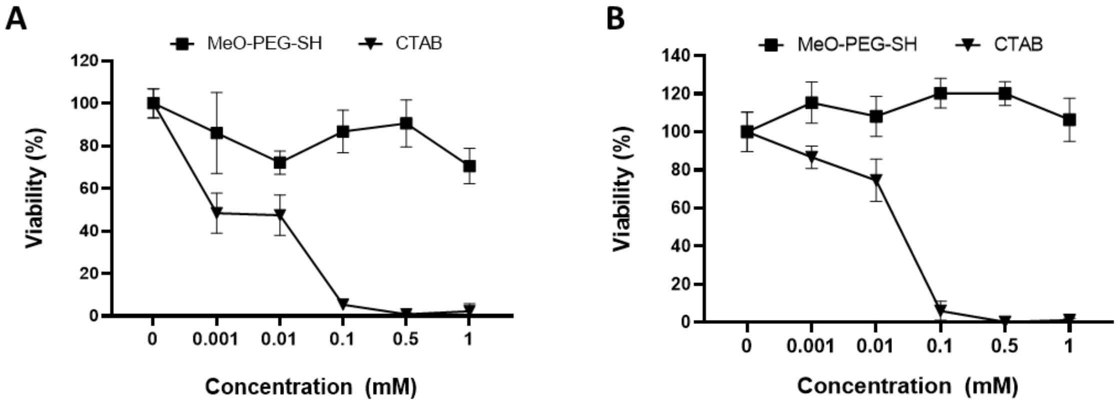

Figure S8. Cytotoxicity of MeO-PEG-SH and CTAB. Cytotoxicity of MeO-PEG-SH and CTAB in **(A)** SN4741 and **(B)** B16-F10 cells measured by the modified LDH assay. Cells were seeded in 96-well plates at 6×10^3 cells/well seeding density then treated with the compounds in the concentration over 0.001 mM and 1 mM concentration range for 24 h. Cell viability was expressed as mean ± SD, n=3-5.

Reference

[1] J. Perez-Juste, L. M. Liz-Marzan, S. Carnie, D. Y. C. Chan, P. Mulvaney, *Adv Funct Mater* **2004**, 14, 571.
